# Bone Health in Aging Men: Does Zinc and Cuprum Level Matter?

**DOI:** 10.3390/biom11020237

**Published:** 2021-02-08

**Authors:** Aleksandra Rył, Tomasz Miazgowski, Aleksandra Szylińska, Agnieszka Turoń-Skrzypińska, Alina Jurewicz, Andrzej Bohatyrewicz, Iwona Rotter

**Affiliations:** 1 Department of Medical Rehabilitation and Clinical Physiotherapy, Pomeranian Medical University, 71-210 Szczecin, Poland; aleksandra.szylinska@gmail.com (A.S.); agi.skrzypinska@gmail.com (A.T.-S.); iwona.rotter@pum.edu.pl (I.R.); 2 Department of Propedeutics of Internal Diseases and Arterial Hypertension, Pomeranian Medical University, 71-252 Szczecin, Poland; miazgowski@interia.pl; 3 Department of Orthopedics, Traumatology and Orthopedic Oncology, Pomeranian Medical University, 71-252 Szczecin, Poland; ala.jurewicz@wp.pl (A.J.); bohatyrewicz@orthopedics.pl (A.B.)

**Keywords:** bone mineral density, Zn/Cu ratio, markers of bone turnover, sex hormones

## Abstract

The aim of this study was to assess the associations of serum and bone zinc (Zn) and cuprum (Cu) with bone mineral density (BMD) and content (BMC), markers of bone turnover, and sex hormones. The study group comprised 144 men treated with total hip replacement due to hip osteoarthritis. We measured total, free, and bioavailable testosterone, estradiol, and sex-hormone-binding globulin (sex hormones), as well as parathyroid hormone, osteocalcin, carboxy terminal collagen crosslinks, and N-terminal propeptide of type I procollagen (markers of bone turnover). Total body BMD, BMC, total and visceral fat, and appendicular skeletal mass (ASM) were measured using dual-energy X-ray absorptiometry. ASM index, and total and visceral fat were positively correlated with BMD. Bone Zn correlated neither with sex hormones nor with bone turnover markers; however, it was positively associated both with BMD and with BMC, while bone Cu (as opposed to serum Cu) was not. In multiple regression, the ASM index, Zn/Cu ratio (in both the serum and the bone), and serum Cu concentration were significantly associated with BMD and BMC after adjustment for age and body mass index (BMI). Our results suggest that the Zn/Cu ratio in both the serum and the bone may exert a significant positive effect on total BMD and BMC.

## 1. Introduction

Bone mass and quality are determined by many genetic, hormonal, and environmental factors. Among hormonal factors, it is well established that sex hormones play a key role in maintaining lifelong skeletal integrity and bone health through a complex variety of endocrine, paracrine, and autocrine actions [1,2]. It is also believed that nutrition is an important modifiable factor in the development and maintenance of bone health and that an adequate intake of macronutrients, mainly proteins, as well as micronutrients, such as vitamins and trace minerals, is essential to support physiological bone remodelling. Among micronutrients, an adequate supply of vitamin D, vitamin K, and calcium was found to be crucial for bone acquisition and maintenance [3,4]. Much less is known about the impact on bone health of other micronutrients, such as zinc (Zn) and copper (Cu).

Zn is a cofactor in metalloenzymes and an important contributor to bone health [4,5]. The skeleton contains a large proportion of the total body Zn, which plays a role in bone growth along the length, regulation of the endocrine axis, and intracellular signaling [5]. It stimulates the proliferation of osteoblasts and bone formation, inhibits osteoclastic bone resorption, and protects osteoblasts against apoptosis. Zinc deficiency effects the integrity of bone tissue and reduces the activity of collagenase, collagen synthesis, and bone mineralization [6]. Zn deficiency negatively correlates with skeletal growth and reduces the synthesis of chondroitin sulfate and the activity of alkaline phosphatase (ALP) [7,8,9]. Studies suggest that impaired osteoid mineralization or cartilage calcification associated with endochondral ossification may contribute to impaired bone growth during Zn deficiency [5]. Cu is a cofactor for lysyl oxidase, an enzyme responsible for the process of crosslinking of collagen fibers and creating crosslinks, the disturbance of which leads to the weakening of the bone structure. Cu reduces bone metabolism by suppressing osteoblasts and osteoclasts. Cu deficiency leads to bone malformations and increases the risk of osteoporosis. The element is significantly involved in the formation of the lysyl oxidase enzyme responsible for the crosslinking of elastin and collagen in the organic matrix of bone, and it was found to be positively associated with tensile strength and bone flexibility [9,10]. It has been suggested that the Zn/Cu ratio rather than serum concentrations of Zn and Cu alone might be a better indicator of bone mineral density (BMD) in the elderly [10], as well as the homeostatic shift from a general systemic growth and reproduction status typical at the juvenile age to a repair and maintenance status aiming to preserve overall health during aging [11]. Trace elements also were studied in various bone disorders including osteoarthritis (OA), a whole-joint disease, in which pathological changes include cartilage destruction, synovial inflammation, osteophyte development, and subchondral bone sclerosis occurring in all tissues of the joint. Although there is no evidence available to date that has clearly indicated that Zn plays a causal role in the pathogenesis of OA, some studies demonstrated the entire series of molecular events in the OA zinc pathway, from zinc influx into cells via transporting protein ZIP8 to cartilage destruction caused by upregulation of the Zn-sensing, matrix-degrading enzymes. In this context, Zn influx might be the essential event that promotes the activation of catabolic cascades in chondrocytes [12,13].

The aim of this study was to assess the associations of Zn and Cu status with total BMD, bone mineral content (BMC), markers of bone turnover, and the sex-hormone milieu. To this end, we measured Zn and Cu content in the bone tissue of the hip joint removed during total hip replacement in patients with hip osteoarthritis.

## 2. Materials and Methods

### 2.1. Study Participants

The study included 144 men treated at the Orthopedics Clinic of the Pomeranian Medical University due to osteoarthritis of the hip joints using the total hip replacement method. Exclusion criteria were diabetes, history of cancer, alcohol abuse, liver or kidney failure, heart failure of class III or IV according to the New York Heart Association (NYHA), and taking drugs that affect bone metabolism, such as mineral supplements, neuroleptics, chemotherapeutic agents, immunosuppressive drugs, corticosteroids, or antidepressants. The study complied with all applicable institutional regulations regarding the ethical use of human volunteers in research and the terms of the Declaration of Helsinki. The Pomeranian Medical University Ethics Committee approved the study protocol, and all participants gave their written consent.

### 2.2. Determination of Sex Hormones and Bone Remodeling Markers

All venous blood samples were obtained after an overnight fast (between 07:00 a.m. and 09:00 a.m.) and stored at −20 °C until processed. The concentrations of total testosterone (TT, normal range: 2.36–9.96 ng/mL), estradiol (E2, normal range for males: 11.2–50.4 pg/mL), sex-hormone-binding globulin (SHBG, normal range: 18–110 nmol/L), and dehydroepiandrosterone sulfate (DHEAS, normal range: 110–470 μg/dL) were determined by ELISA (DRG Medtek, Warsaw, Poland). The free testosterone (FT, normal range: 8.9–45.5 pg/mL) level was calculated using the following formula developed by Vermeulen:

FT = (TT − N − SHGB + √((N + SHGB − TT)^2^ + 4NT))/2N,

where N = 0.5217 × albumin concentration + 1 [14]. The bioT concentration was calculated using the following formula: bioT = FT concentration (ng/dL) + albumin concentration (g/L) [15]. The sensitivity of the methods for total testosterone was 0.083 ng/mL with the limit of quantification (LOQ) of 0.249 ng/mL; the intra- and inter-assay coefficients of variation (CV) were 3.28% and 6.71%, respectively. The sensitivity of the E2 assay was 9.714 pg/mL with the LOQ of 29.142 pg/mL; inter- and intra-assay CVs were 6.72% and 2.71%, respectively. The sensitivity of SHGB assay was 0.001 nmol/L with the LOQ of 1.65 nmol/L; inter- and intra-assay CVs were 3.0% and 7.2%, respectively. For the DHEAS assay, inter-assay CV was 8.9% and intra-assay CV was 10.5%.

The following markers of bone turnover were measured: osteocalcin (OC, normal range: 5–25 ng/mL), parathyroid hormone (PTH, normal range: 10–60 pg/mL), carboxy-terminal collagen I crosslinks (CTX-I, normal range: 0.115–0.748 ng/mL), and human procollagen I aminoterminal propeptide (PINP, normal range: 85.55–2028.75 ng/mL).

### 2.3. Determination of Serum and Bone Zn and Cu

Extracted serum samples and bone samples were frozen at −80 °C until processed. Serum and bone Zn and Cu were analyzed using inductively coupled plasma optical emission spectrometry (iCAP™ 7400 ICP-OES Analyzer; Thermo Fisher Scientific, Waltham, MA, USA), which is an established and powerful technique for the analysis and quantitation of trace elements in both liquid and solid samples. Analysis was performed in radial and axial mode. Serum samples were thawed at room temperature and digested using the oven digestion system CEM MARS 5. Bone samples also were thawed at room temperature and then dried overnight at 80 °C to constant weight after cleaning all the adherent tissues. The bones were ground into powder in a porcelain mortar and mineralized using the CEM MARS 5 system. The bone tissue (at least 0.1 g of weight) was then allowed 30 min of pre-reaction time in the clean hood. After completion of the pre-reaction time, 1 mL of non-stabilized 30% H_2_O_2_ was added. Then, the samples were placed in special Teflon vessels and heated in a microwaved digestion system for 35 min in 180 °C. At the end of the digestion, all samples were removed from the microwave and allowed to cool to room temperature. Blank samples were prepared by adding concentrated nitric acid to tubes without sample and subsequently diluted in the same manner. Multi-element calibration standards (ICP multi-element standard solution IV; Merck, Darmstadt, Germany) were prepared with different concentrations of inorganic elements in the same manner as the blanks and samples. Deionized water (Direct Q UV, Merck, Darmstadt, Germany; approximately 18.0 MΩ) was used for the preparation of all solutions. For the calibration of bone samples, we used Merck, Darmstadt, Germany), which consists of steamed bone meal that was sieved and blended to a high degree of homogeneity (a unit of SRM 1486 consists of approximately 50 g of bone meal; certified mass fraction values for Zn = 147 ± 16 mg/kg and 0.8 mg/kg for Cu). Quality control for determination of both trace elements in serum was applied by analyzing the SRM (8414 NIST Bovine muscle, Gaithersburg, MD, USA) using Instrumental Neutron Activation Analysis. The accuracy of the measurements (expressed as % recovery) and precision (% coefficient of variance) were in the range of 93–103% and 92–112%, whereas the % coefficient of variation was 3.62% and 5.17% for Zn and Cu, respectively. The wavelengths were 213.856 nm and 324.754 nm for Zn and Cu, respectively.

Serum molar concentrations of Zn and Cu were calculated according to the following formula: molar concentration (nmol/g) = concentration (nmol/g) × 1000/atomic weight (g/mol) [16], with normal ranges: 11–18 µmol/L and 12.6–25.2 µmol/L for Zn and Cu, respectively.

### 2.4. Densitometry

Dual-energy X ray absorptiometry (DXA) (Lunar Prodigy Advance; GE Healthcare; Madison, WI, USA) was used to assess total body BMD and body composition, including BMC, lean mass (a surrogate measure of muscle mass), and total fat mass, using the automatic whole-body scan mode. From lean mass, appendicular skeletal mass (ASM) was calculated as the sum of the muscle mass of both arms and legs, assuming that all nonfat and nonbone tissue is skeletal muscle. From ASM and height, the ASM index was calculated using the following formula: ASMI = ASM/height^2^ [17,18], with sex-specific normal values: >7.26 kg/m^2^ [19]. Visceral fat was assessed using a device-specific CoreScan (GE Healthcare, Madison, WI, USA) application, which computes visceral fat mass and volume by subtracting subcutaneous fat from total fat in the android region of interest. All scans were performed and analyzed by a single trained technician. Instrument quality control was performed on a regular basis (at least 3 times per week and additionally at the day of the patient’s assessment) using the manufacturer’s block phantom and the Hologic spine phantom (Hologic Inc., Marlborough, MA, USA) scanned at least once a week. There was no significant drift in calibration within the entire study period.

### 2.5. Statistical Analysis

Quantitative variables were presented as means (ranges) and standard deviation (SD). Data were checked for normality using the Shapiro–Wilk test. In the case of normal distribution, means were compared using Student’s *t*-test; otherwise, the nonparametric Mann–Whitney U-test was used. The relationship between pairs of quantitative variables was analyzed using Pearson’s linear correlation coefficient. Multiple linear regression models with the Bonferroni correction were adjusted for age and body mass index (BMI). A receiver operating characteristic (ROC) curve analysis was used to assess the accuracy of the Zn/Cu ratio. The accuracy was measured using the area under the curve (AUC) with a 95% confidence interval (CI). To determine the specific cutoff point for Zn/Cu ratio, the score with the highest combination of sensitivity and specificity (Youden’s index) was considered optimal. Statistical analyses were performed using Statistica (version 12.0, StatSoft Poland, Cracow, Poland). The significance level was set at *p* ≤ 0.05. The statistical power of the study with 144 subjects was sufficient to detect with 80% probability true associations with strength corresponding to correlation coefficient or standardized beta coefficient equal to ±0.24 at a statistical significance level of *p* = 0.05.

## 3. Results

### 3.1. Characteristics of Study Participants

The study comprised 144 men aged 60–75 years. Among them, 97 had concomitant hypertension, 41 had benign prostatic enlargement, 12 had hyperuricemia, eight had coronary heart disease, and three had prior stroke; only four were current smokers. Baseline characteristics are shown in Table 1. In 17 (12%) patients, the BMI was normal, 68 (47%) were overweight (BMI between 25.0 and 29.9 kg/m^2^), and 59 (41%) were obese. Serum Zn/Cu ratio was 1.4, and that in the bone was 171.7. The mean values of ASMI, as well as sex hormones and markers of bone turnover, were within normal ranges. However, up to 50% patients had elevated E2 and 41% had low osteocalcin levels (Table 2).

### 3.2. Associations of Zn and Cu with Study Variables

Correlations of Zn and Cu with age, BMI, sex hormones, bone turnover markers, and densitometric parameters are shown in Table 3. Serum but not bone Cu positively correlated with age. It was also inversely correlated with FT, bioT, BMC, and ASMI. In contrast to Cu, serum Zn was positively correlated with FT and bioT, but not with age. Interestingly, the Zn/Cu ratio in serum was positively correlated with FT, bioT, and BMC, and these associations were much stronger than those of serum Zn and Cu alone. In the bone tissue, Zn content correlated neither with sex hormones nor with bone turnover markers; however, it was positively associated both with total body BMD and with BMC. In contrast to Zn, the bone content of Cu was not correlated with testosterone and bone mass. However, the Zn/Cu ratio showed a significant positive association with BMC but not BMD. Interestingly, serum levels of both trace elements did not correlate with their content in the femoral neck (*r* = 0.019 and *r* = −0.042 for Zn and Cu, respectively).

Next, we tested the correlates of total body BMD and BMC. As shown in Table 4, age, total fat, and visceral fat were positively correlated, but the strongest association was found with ASMI (*r* = 0.504 and *r* = 0.473 for total BMD and BMC, respectively). Rather unexpectedly, markers of bone turnover correlated neither with BMD nor with BMC. Similarly, they also were not correlated with sex hormone levels.

**Figure 1:**
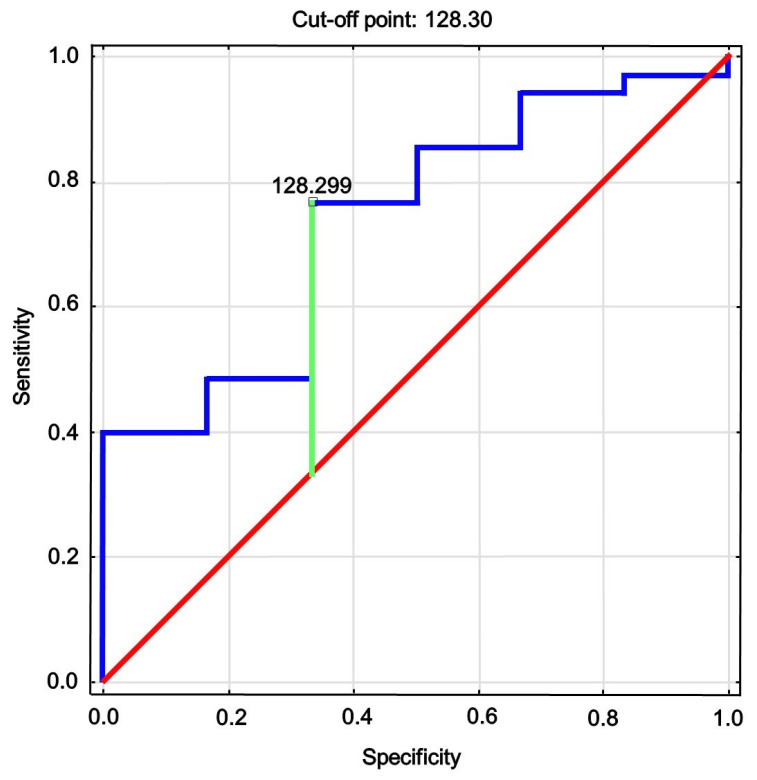
Receiver operating characteristics of bone Zn/Cu ratio for identifying total body BMD > −1 SD.

In multiple regression, only ASMI, the Zn/Cu ratio (both in serum and in bone), and serum Cu concentration were significantly associated with total body BMD and BMC after adjustment for age and BMI (Table 5). In the AUC analysis (Table 6), the Zn/Cu ratio in bone was a moderate predictor for normal total body BMD defined as total body *z*-score >−1 SD. However, the value of Youden’s index, which was used as a measure of quality for optimal cutoff, was relatively low. ROC curve analysis of the bone Zn/Cu ratio for prediction of normal BMD is shown in Figure 1.

## 4. Discussion

The study was performed on patients aged 60–75 years with advanced OA and frequent coincidence of overweight/obesity. This is a typical cohort for hip replacement in terms of age, comorbidities, and indications for this procedure [20,21]. This is the first study to compare the association of serum and bone Zn and Cu levels with DXA-derived total bone mass and bone mineral content. Previous studies suggested a high serum Cu/Zn ratio (due to the decrement of serum Zn and/or increment of serum Cu) might be a marker of inflammation [22], risk for cardiovascular death [23], malignancy [24], and all-cause mortality in older subjects [22]. We found the Zn/Cu ratio (we used the reversed ratio instead of the Cu/Zn ratio used in studies cited above) to be positively associated with bone density and mineral content, suggesting the ratio might be an important indicator of bone status in men. When both trace minerals were tested separately, serum Cu concentration was negatively associated with both BMC (β = −0.349) and BMD (β = −0.324), while Zn concentration was not. Interestingly, this association remained significant even after the adjustment for age and BMI. Although serum Zn and Cu levels appear to be only slightly affected by nutrition (except for severe deficiency or supplementation periods [25,26]), serum levels of Cu increase while Zn decrease with age [11]. It has been suggested that these changes might be driven by age-related increase in the level of oxidative stress [11] and low-grade chronic proinflammatory states, as well as hormonal changes [11,27]. A similar, age-dependent pattern was observed also in our study but exclusively in the relation to Cu. Additionally, we found serum Cu levels to be negatively correlated with the level of testosterone while the correlation between Zn and testosterone was positive, suggesting that changes in Zn and Cu in men might be triggered by a decline in free and bioavailable testosterone with age. Earlier studies on the relationship between serum Cu and BMD measured at various sites have yielded conflicting results as the associations were positive [28,29], negative [10,30], or neutral [31,32,33]. In turn, the association between serum Zn and BMD was positive in several reports [34,35,36]; however, in the recent meta-analysis, serum zinc level did not show a significant difference in overall bone turnover–related conditions, such as osteoporosis, osteopenia, fracture, or postmenopause from control groups, and it was not correlated with lumbar spine and hip BMD [37].

We found in bone specimens that Zn and Cu contents were correlated neither with age nor with sex hormones, suggesting other mechanisms to be involved in the accumulation of these minerals in bones. Similarly, Zn and Cu did not correlate with markers of bone turnover. Moreover, serum levels of both trace elements did not correlate with their content in the femoral neck. The latter finding may suggest a different pathway of Zn and Cu accumulation in bones which is independent from their serum concentrations, at least if they are within normal ranges. Hence, it can be speculated that, if both elements are given as dietary supplements, their positive effects on overall bone health could be questionable. As opposed to the serum levels, bone Zn showed positive correlations with BMD and BMC, but the association became insignificant after controlling for confounders. On the other hand, the bone Zn/Cu ratio, similarly to the serum ratio, was positively associated with bone mineral density and content, confirming that the balance between these two elements both in serum and in bone appears to be important for bone maintenance. From a practical point of view, in further studies evaluating the content of Zn and Cu in bones, we suggest the use of the Zn/Cu ratio rather than the opposite Cu/Zn ratio, as bone content of Zn is much higher than Cu (in this study: 2909 µmol/kg vs. 23.5 µmol/kg for Zn and Cu, respectively).

Another finding of this study refers to the association between body composition and bone mass. We found that visceral fat and total body fat were significantly positively correlated with total body BMD and BMC. However, after adjusting for confounders, these associations were no longer significant. In our previous study, total fat was a significant predictor of lumbar spine and total hip BMD, and visceral fat of lumbar spine BMD in older men [38]. On the other hand, in the study of Zhu et al. [39], visceral fat was negatively associated with total body, lumbar spine, and femoral neck BMD in men aged 58 ± 6 years (a cohort younger than ours), suggesting that excess visceral fat may be deleterious to bone, especially in males. Visceral fat measured by computed tomography was also negatively correlated with hip and spine BMD in Korean adults [40], but it is uncertain whether these results can be extrapolated to the Caucasian population, in light of the ethnic differences in body composition and BMI. Regardless of these inconsistences, an important confounding factor to consider when examining associations between visceral fat and BMD is BMI, which, in the vast majority of earlier studies [39,40,41], as well as in this study, was positively correlated with both visceral fat and BMD. In contrast to visceral fat, a positive impact of skeletal muscle mass on bone health has been well established [39,42,43]. Our results strongly support these findings as ASMI was positively correlated with both BMD and BMC, and this association remained significant after adjustment for age and BMI.

We did not find the associations of sex hormones with bone outcomes. Generally, in contrast to menopause, a decline in testicular function in men can be initiated in any time point during or after middle age and a decrease in testosterone level is usually gradual and much less overt in comparison with postmenopausal deficit of estrogens [1,2,44]. In our study population, the mean values of testosterone were within normal ranges, and only 3% of patients had slightly decreased FT and bioT. On the other hand, 49% had high E2 level. However, bone loss in men is mainly related to relative estrogen deficiency, while androgens have a minor role. It has been demonstrated in adult men without genetic diseases involving estrogen pathways that E2 can be deleterious to bone mass but only when serum levels are below a critical threshold settled between 20 and 25 pg/mL [45] and, thus, much lower than in our cohort. Therefore, bone loss in men usually can occur in patients with very low testosterone (hypogonadism) and normal or low testosterone combined with low E2 [44]. In addition, 88% of our patients were overweight or obese, and, in the human male, E2 derives from the aromatization of the androgens by means of the enzymatic complex of aromatase, which is widely expressed in the adipose tissue. Through this mechanism, E2 can exert a protective effect on bone mass.

Our study had some limitations. Firstly, it was an observational study, and it cannot be assumed that the associations among trace elements, fat distribution, and bone mass were causal. Secondly, we did not evaluate the level of physical activity, alcohol consumption, dietary and supplementary intake of calcium, Zn, and Cu, and vitamin D concentration, which are known to affect BMD/BMC. Lastly, the current study was performed on patients with OA and, hence, the generalization of our findings to healthy individuals or patients with other bone disorders and other medical conditions should be exercised with caution.

In conclusion, in aging men with OA, the Zn/Cu ratios in both the serum and the bone but not visceral fat, sex hormones, and markers of bone turnover were positively associated with total body BMD and BMC after controlling for confounders.

## Figures and Tables

**Table 1:** Baseline characteristics of study participants.

	Mean	SD	Range
Anthropometric parameters			
Age (years)	67.05	4.35	60.00–75.0
Weight (kg)	90.63	15.17	51.00–130.0
BMI (kg/m^2^)	29.61	3.13	20.42–42.91
Sex hormones			
Total testosterone (ng/mL)	4.76	2.12	0.52–12.9
Free testosterone (pg/mL)	9.19	3.34	1.61–23.45
Bioavailable testosterone (ng/dL)	1.94	0.92	0.13–5.06
Estradiol (pg/mL)	82.65	41.0	20.9–236.0
SHBG (nmol/L)	52.49	41.6	4.06–215.5
DHEAS (µg/dL)	81.03	66.32	9.45–407.4
Markers of bone turnover			
CTX-I (ng/mL)	0.44	0.21	0.06–1.80
PTH (pg/mL)	36.49	22.32	2.70–161.3
Osteocalcin (ng/mL)	6.33	4.11	0.20–29.94
PINP (ng/mL)	938.1	917.7	5.82–3689
Serum Zn and Cu			
Zn (µmol/L)	21.71	6.07	3.5–46.55
Cu (µmol/L)	15.66	3.32	3.81–30.51
Zn/Cu ratio	1.42	0.46	0.49–3.90
Bone Zn and Cu			
Zn (µmol/kg)	2908.7	841.63	1041–5301
Cu (µmol/kg)	23.48	16.01	2.64–117.2
Zn/Cu ratio	171.72	115.41	29.53–611.9
Densitometry			
Total body BMD (g/cm^2^)	1.25	0.15	0.99–1.56
Total body BMD (z-score)	0.44	1.25	−2.2–2.1
BMC (g)	3051.2	395.65	2086–4012
Fat (kg)	31.57	6.986	13.89–47.27
Fat (%)	35.58	5.72	10.02–44.71
Visceral fat (kg)	2.65	0.92	0.42–4.80
Lean mass (kg)	55.04	6.76	39.94–74.59
ASMI (kg/m^2^)	8.08	1.0	6.32–11.8

BMI, body mass index; SHBG, sex-hormone-binding globulin; DHEAS, dehydroepiandrosterone sulfate; CTX-I, carboxy-terminal collagen I crosslinks; PINP, human procollagen I N-terminal peptide; PTH, parathyroid hormone; BMD, bone mineral density; BCM, bone mineral content; ASMI, appendicular skeletal muscle mass index.

**Table 2:** Frequency of abnormal values of sex hormones and bone turnover markers.

	Normal	Low	High
Sex hormones			
Total testosterone	132 (92%)	12 (8%)	
Free testosterone	140 (97%)	4 (3%)	
Bioavailable testosterone	139 (96.5%)	5 (3.5%)	
Estradiol	73 (51%)		71 (49%)
SHGB	111 (77%)	18 (12.5%)	15 (10.5%)
Markers of bone turnover			
Osteocalcin	85 (59%)	59 (41%)	
Parathyroid hormone	130 (90%)		14 (10%)
CTX-I	126 (87.5%)		18 (12.5%)
PINP	119 (82.5%)	7 (5%)	18 (12.5%)

**Table 3:** Correlations of Zn and Cu with age, BMI, sex hormones, bone turnover markers, and densitometric parameters.

	Serum			Bone		
	Zn/Cu Ratio	Zn	Cu	Zn/Cu Ratio	Zn	Cu
Age	−0.223	−0.087	**0.502**	−0.153	−0.101	0.231
	*p* = 0.253	*p* = 0.659	*p* = 0.006	*p* = 0.435	*p* = 0.607	*p* = 0.834
BMI	0.006	−0.086	−0.295	0.070	0.142	0.0041
	*p* = 0.976	*p* = 0.662	*p* = 0.127	*p* = 0.720	*p* = 0.468	*p* = 0.834
TT	0.268	0.175	−0.189	0.237	−0.009	−0.275
	*p* = 0.168	*p* = 0.373	0.333	*p* = 0.224	*p* = 0.961	*p* = 0.157
FT	**0.730**	**0.543**	**−0.527**	0.117	0.161	−0.178
	*p* < 0.001	*p* = 0.002	*p* = 0.004	*p* = 0.552	0.411	*p* = 0.365
bioT	**0.666**	**0.548**	**−0.445**	0.179	0.185	−0.255
	*p* < 0.001	*p* = 0.003	*p* = 0.018	*p* = 0.360	*p* = 0.345	*p* = 0.189
E2	0.204	0.245	−0.044	0.096	0.009	−0.305
	*p* = 0.296	*p* = 0.208	*p* = 0.822	*p* = 0.626	*p* = 0.961	*p* = 0.114
PINP	0.294	0.265	−0.192	0.171	0.127	−0.216
	*p* = 0.128	*p* = 0.172	*p* = 0.327	*p* = 0.382	*p* = 0.517	*p* = 0.811
SHGB	−0.189	−0.172	0.172	0.081	−0.165	−0.047
	*p* = 0.333	*p* = 0.382	*p* = 0.380	*p* = 0.681	*p* = 0.401	*p* = 0.811
DHEAS	0.348	0.343	−0.114	0.227	0.122	−0.233
	*p* = 0.069	*p* = 0.074	*p* = 0.563	*p* = 0.244	*p* = 0.534	*p* = 0.231
CTX-I	−0.307	−0.300	0.140	−0.099	0.050	0.039
	*p* = 0.111	*p* = 0.120	*p* = 0.477	*p* = 0.614	*p* = 0.800	*p* = 0.843
PTH	−0.041	−0.030	−0.013	0.017	0.368	−0.142
	*p* = 0.832	*p* = 0.877	*p* = 0.945	*p* = 0.930	*p* = 0.054	*p* = 0.471
OC	−0.169	−0.145	0.137	0.013	0.310	−0.040
	*p* = 0.389	*p* = 0.460	*p* = 0.486	*p* = 0.947	*p* = 0.108	*p* = 0.840
BMD	0.339	0.225	−0.365	0.299	**0.400**	−0.240
	*p* = 0.078	*p* = 0.249	*p* = 0.055	*p* = 0.122	*p* = 0.035	*p* = 0.219
BMC	**0.475**	0.319	**−0.413**	**0.375**	**0.470**	−0.244
	*p* = 0.011	*p* = 0.098	*p* = 0.029	*p* = 0.049	*p* = 0.012	*p* = 0.211
ASMI	0.205	0.002	**−0.535**	0.293	0.339	−0.205
	*p* = 0.293	*p* = 989	*p* = 0.003	*p* = 0.130	0.077	*p* = 0.295

BMI, body mass index; TT, total testosterone; FT, free testosterone; bio T, bioavailable testosterone; SHBG, sex-hormone-binding globulin; E2, estradiol; DHEAS, dehydroepiandrosterone sulfate; CTX-I, carboxy-terminal collagen I crosslinks; PINP, human procollagen I N-terminal peptide; PTH, parathyroid hormone; OC, osteocalcin; BMD, bone mineral density; BCM, bone mineral content; ASMI, appendicular skeletal muscle mass index; bold, statistically significant results.

**Table 4:** Correlations of BMD and BMC with age, BMI, sex hormones, ASMI, and fat.

	Bone Mineral Density		Bone Mineral Content	
	*R*	*p*-Value	*R*	*p*-Value
Age	−0.294	0.077	−0.090	0.595
BMI	**0.531**	0.001	**0.477**	0.003
Total testosterone	0.061	0.718	0.196	0.243
Free testosterone	0.152	0.367	0.179	0.287
Bioavailable testosterone	0.181	0.283	0.220	0.190
Estradiol	−0.085	0.614	−0.186	0.270
SHBG	−0.140	0.407	0.008	0.960
DHEAS	0.146	0.389	0.105	0.536
Total fat	**0.397**	0.015	**0.403**	0.013
Visceral fat	**0.335**	0.015	**0.356**	0.030
ASMI	**0.504**	0.001	**0.473**	0.003

Bold, statistically significant results.

**Table 5:** Regression analysis adjusted for age and BMI. CI, confidence interval.

	Total Bone Mineral Density				Bone Mineral Content			
	Beta	*p*-Value	95% CI		Beta	*p*-Value	95% CI	
			Low	High			Low	High
Sex hormones								
FT	0.150	0.364	−0.182	0.484	0.268	0.122	−0.075	0.613
bioT	0.156	0.334	−0.159	0.168	0.280	0.097	−0.053	0.614
E2	−0.115	0.436	−0.410	0.180	−0.183	0.212	−0.475	0.108
Serum Zn and Cu								
Zn	0.108	0.443	−0.172	0.387	0.115	0.414	−0.165	0.394
Cu	**−0.324**	0.018	−0.590	−0.058	**−0.349**	0.010	−0.612	−0.085
Zn/Cu ratio	**0.286**	0.023	0.041	0.532	**0.299**	0.030	0.031	0.567
Bone Zn and Cu								
Zn	0.286	0.070	−0.024	0.597	0.207	0.195	−0.110	0.523
Cu	−0.253	0.111	−0.566	0.061	−0.255	0.108	−0.568	0.058
Zn/Cu ratio	**0.330**	0.035	0.024	0.636	**0.346**	0.027	0.042	0.650
Densitometric parameters								
Total fat	0.011	0.955	−0.386	0.408	0.148	0.472	−0.265	0.562
Visceral fat	0.041	0.786	−0.262	0.344	0.106	0.501	−0.210	0.423
ASMI	**0.334**	0.035	0.024	0.644	**0.352**	0.034	0.027	0.676

Bold, statistically significant results.

**Table 6:** Area under the curve (AUC) value and cutoff for Zn/Cu ratio for prediction normal BMD.

AUC	95% CI	*p*-Value	Cutoff	Sensitivity	Specificity	Youden’s Index
0.738	0.537; 0.939	0.020	128.3	0.78	0.34	0.44
